# Gene Expression Profiles Controlled by the Alternative Splicing Factor Nova2 in Endothelial Cells

**DOI:** 10.3390/cells8121498

**Published:** 2019-11-23

**Authors:** Elisa Belloni, Anna Di Matteo, Davide Pradella, Margherita Vacca, Christopher D. R. Wyatt, Roberta Alfieri, Antonio Maffia, Simone Sabbioneda, Claudia Ghigna

**Affiliations:** 1 Istituto di Genetica Molecolare, “Luigi Luca Cavalli-Sforza”, Consiglio Nazionale delle Ricerche, via Abbiategrasso 207, 27100 Pavia, Italy; elisa.belloni@igm.cnr.it (E.B.); anna.dimatteo@igm.cnr.it (A.D.M.); davide.pradella@igm.cnr.it (D.P.); margheritavacca96@gmail.com (M.V.); roberta.alfieri@igm.cnr.it (R.A.); antonio.maffia@igm.cnr.it (A.M.); sabbioneda@igm.cnr.it (S.S.); 2 Centre for Biodiversity and Environment Research, University College London, Gower Street, London WC1E 6BT, UK; 3 Centre for Genomic Regulation (CRG), The Barcelona Institute of Science and Technology, Dr Aiguader 88, 08003 Barcelona, Spain; 4 Universitat Pompeu Fabra, Plaça de la Mercè, 10-12, 08002 Barcelona, Spain

**Keywords:** Nova2, alternative splicing, angiogenesis, vascular development, post-transcriptional regulation

## Abstract

Alternative splicing (AS) plays an important role in expanding the complexity of the human genome through the production of specialized proteins regulating organ development and physiological functions, as well as contributing to several pathological conditions. How AS programs impact on the signaling pathways controlling endothelial cell (EC) functions and vascular development is largely unknown. Here we identified, through RNA-seq, changes in mRNA steady-state levels in ECs caused by the neuro-oncological ventral antigen 2 (Nova2), a key AS regulator of the vascular morphogenesis. Bioinformatics analyses identified significant enrichment for genes regulated by peroxisome proliferator-activated receptor-gamma (Ppar-γ) and E2F1 transcription factors. We also showed that Nova2 in ECs controlled the AS profiles of *Ppar-γ* and E2F dimerization partner 2 (*Tfdp2*), thus generating different protein isoforms with distinct function (Ppar-γ) or subcellular localization (Tfdp2). Collectively, our results supported a mechanism whereby Nova2 integrated splicing decisions in order to regulate Ppar-γ and E2F1 activities. Our data added a layer to the sequential series of events controlled by Nova2 in ECs to orchestrate vascular biology.

## 1. Introduction

Alternative splicing (AS), which generates different mature transcripts (mRNAs) from a single gene, is recognized to play a prominent role in expanding the coding potential of the human genome through the generation of specialized protein variants required in precise cellular processes, tissues, or developmental stages [1]. AS events affect nearly all human protein-coding genes [2,3] and are directed by a number of splicing regulatory factors (SRFs) that bind *cis*-regulatory sequences within the nascent pre-mRNA and promote inclusion or skipping of specific AS exons.

Mutations in splicing *cis*-regulatory sequences and/or altered expression of SRFs are features of cancer cells driving AS changes involved in activation of oncogenes, inhibition of tumor suppressors, as well as the acquisition of drug resistance [4,5,6,7]. In addition, a number of SRFs function themselves as oncogenes [8,9] or tumor suppressors [10,11], generating cancer-associated AS proteins involved in almost all aspects of tumor cell biology [4]. Indeed, aberrant AS is now recognized as another hallmark of cancer [12,13], and cancer-associated AS isoforms can be used to stratify patients for cancer diagnosis or as predictive biomarkers of prognosis [14,15]. Furthermore, their targeting can be exploited to improve the development of novel anti-cancer therapies [6,12].

Angiogenesis, the formation of new blood vessels from the existing vasculature, plays fundamental roles during embryonic development, but it is also essential to sustain several pathological conditions, including cancer progression [16,17]. In contrast to the well-known mechanisms that control angiogenesis at the transcriptional level, the information regarding the role of AS programs in regulating endothelial cell (EC) functions is still limited. This is even more evident in the context of cancer vasculature in which tumor ECs express a number of peculiar AS isoforms (absent or expressed at low levels in adult normal tissues) [18,19,20,21,22], which have been investigated in clinical studies to develop anti-angiogenic cancer treatments [23,24]. However, for most of these AS variants, their biological roles in tumor angiogenesis—as well as the SRFs responsible for their production—are still largely unknown. Thus, a better understanding of the AS role during EC differentiation and morphogenesis could lead to a deeper comprehension of vascular system development and also provide a new perspective to understand the aberrant phenotype of tumor blood vessels.

In the past, we discovered that neuro-oncological ventral antigen 2 (Nova2), a tissue-restricted SRF previously identified and characterized for its important functions in the neurons of the central nervous system [25,26,27], was also expressed in the vascular endothelium and played a relevant role in vascular morphogenesis [28]. From genome-wide approaches and histological analysis of *Nova2* mutant animals, we demonstrated that Nova2 acted as a post-transcriptional regulator of the molecular mechanisms involved in the establishment of EC polarity and the organization of the vascular lumen during blood vessels development [28]. Very recently, another group found that the Nova2-mediated splicing controlled the Erk signaling downstream of VEGFC-Flt4 in order to regulate lymphatic endothelial cells’ specification [29]. Notably, Nova2 was recently reported to be upregulated in the vasculature of colorectal and ovarian cancers [30,31], and high Nova2 expression correlates with shorter overall survival of ovarian cancer patients [31].

Structurally, Nova2 contains three hnRNP K homology (KH) RNA binding domains, and it directly binds its pre-mRNA targets at the level of YCAY (Y = C/U) motifs localized in close proximity of the regulated AS exons [25]. The position of Nova2 binding sites predicts the outcome of the splicing reaction following the rule that Nova2 promotes exon skipping when bound to the exonic or upstream intronic YCAY clusters, while it stimulates exon inclusion when interacting with downstream intronic motifs [25,32]. Finally, in addition to its important role in AS regulation, and similar to many other SRFs, Nova2 shuttles between the nucleus and cytoplasm, suggesting that it could control transport, localization, and stability of a number of mRNA targets [33,34].

In order to better characterize Nova2 functions in vascular endothelium, we investigated changes in the whole transcriptome after its knockdown in ECs, thus identifying novel molecular pathways regulated by this SRF, which could play a relevant role to orchestrate EC biology and vascular development.

## 2. Materials and Methods

### 2.1. Cell Culture

HeLa cells (ATCC, CCL-2) were grown in DMEM-High Glucose (Euroclone, Pero, Italy) supplemented with 10% FBS (Euroclone), 4 mM L-glutamine (Lonza, Basel, Switzerland), and 100 U/L penicillin/streptomycin (Euroclone). Mouse endothelial cells (moEC), previously referred as vascular endothelial (VE) cadherin-positive ECs and described in [28,31,35,36], were cultured in DMEM-High Glucose (Lonza) with 10% FBS, 2 mM L-glutamine (Lonza), 100 U/L penicillin/streptomycin (Euroclone), 1 mM sodium pyruvate (Sigma–Aldrich, Merck, Darmstadt, Germany), 25 mM HEPES (Sigma–Aldrich), 100 µg/mL heparin (from porcine intestinal mucosa, Sigma–Aldrich), and 50 μg/mL EC growth supplement (ECGS from bovine pituitary gland, Sigma–Aldrich). Before seeding, plates were coated with 0.1% porcine gelatin (Difco) and incubated overnight at 37 °C. Cells were maintained in a humidified, 5% CO_2_ atmosphere at 37 °C. For VEGF stimulation, moEC were grown in a serum-starved (0.2% FBS) medium, without ECGS supplementation, for 2 h prior to treatment with recombinant murine VEGF-165 (100 ng/mL, PeproTech, EC Ltd., London, UK) for 24 h.

### 2.2. Plasmids and Transfection

The cDNAs, encoding mouse wild-type full-length Tfdp2 (ENSMUST00000188750.6) and Tfdp2-∆7 (deleted of exons 7) isoforms, were generated by using RT from moEC and cloned into the pEGFP-C1 vector (Clontech, Takara Bio Europe, Saint-Germain-en-Laye, France) in-frame with the EGFP sequence. The primers used for PCR are listed in Supplementary Table S2. All PCR products were verified by sequencing. HeLa cells for the analysis of Tfdp2 localization were grown on microscope slides and transiently transfected with Lipofectamine 3000 (Life Technologies, Thermo Fisher Scientific, Waltham, MA, USA) according to the manufacturer’s protocol. After 24 h, cells were fixed with 4% PFA for 20 min. Slides were washed in PBS, and nuclei counterstained with DAPI solution (0.2 mg/mL, Sigma–Aldrich). The localization of pEGFP-Tfdp2 variants was analyzed through epifluorescence microscopy (Optical Microscope Olympus IX71, Olympus, Tokyo, Japan). For the analysis of E2F1 downstream target activation, HeLa cells (in 100-mm Petri dishes) were transiently transfected with GFP-fusion Tfdp2 constructs or empty vector. 24 h post-transfection, GFP-positive cells were sorted with a cell sorter (S3e, Biorad, Hercules, CA, USA) with gates designed for equal mean fluorescence intensity. Total RNA from the sorted cells was extracted and analyzed by RT-qPCR.

### 2.3. SiRNA-Mediated RNA Interference

For transient depletion experiments, moEC were transfected with siRNAs against mouse *Rbfox2* gene or non-silencing control (SMARTpool: Rbfox2 L-051552-01, Life Technologies; ON-TARGETplus non-targeting pool D-001810-10, Dharmacon, Lafayette, CO, USA) and the Lipofectamine RNAiMax kit (Life Technologies) in accordance with the manufacturer’s instructions. To achieve optimal knockdown efficiency, two subsequent transfections with 70 nM and 40 nM, respectively, of each siRNA oligo were performed with a 24 h interval, and ECs were analyzed 24 h after the second transfection.

### 2.4. Lentivirus Production and Transduction

MoEC were transduced with lentiviral vectors carrying human HA-tagged *NOVA2* cDNA (pLenti-GIII-CMVhumanNOVA2-HA, THP Medical Products, Wien, Austria) or shRNA against the mouse *Nova2* gene (GIPZ shRNAs from Open Biosystems, Huntsville; AL, USA), as described in [28]. After 48 h of infection, the medium was refreshed, and puromycin selection (3 μg/mL, Santa Cruz Biotechnology, Dallas, TX, USA) was started and continued until all non-infected control cells died (typically, five days). Since we reported that Nova2 expression was regulated by EC density [28], for the analysis of the expression levels, differentially expressed genes (DEGs) and the AS analysis of Nova2 target genes (*Ppar-γ* and *Tfdp2*), Nova2 depleted moECs were grown as confluent (500,000 cells in 35-mm Petri dishes), whereas Nova2 overexpressing moEC were grown as sparse (500,000 cells in 100-mm Petri dishes).

### 2.5. Immunoblot Analysis

Total proteins were extracted by using Laemmli buffer (4% SDS, 16% glycerol, 40 mM Tris-HCl pH 6.8), and cell lysates were quantified with Pierce BCA protein assay kit (Thermo Fisher Scientific). Proteins (20 μg/lane) were resolved by SDS-PAGE, transferred to nitrocellulose membranes (Protran, Whatman). Proteins of interest were visualized using specific antibodies, followed by peroxidase-conjugated secondary antibodies. The following primary antibodies were used: anti-Nova2 C-16 (1:200, Santa Cruz Biotechnology), anti-α-Tubulin (1:50,000, Sigma–Aldrich), anti-Vinculin (1:10,000, Cell Signaling, Leiden, The Netherlands), anti-Rbfox2 (1:2000, Bethyl Laboratories, Montgomery, TX, USA), anti-HA High Affinity (1:1000, Roche, Basel, Switzerland), anti-GFP (1:1000, Millipore, Merck, Darmstadt, Germany), anti-E2F1 (1:200, Santa Cruz Biotechnology). Chemiluminescent signal was detected by using ECL LiteAblot Plus/Extended/Turbo (Euroclone) and acquired by ImageQuant LAS 500 chemiluminescence CCD-camera (GE Healthcare, Chicago, IL, USA).

### 2.6. Co-Immunoprecipitation (Co-IP)

HeLa cells (in 100-mm Petri dishes) were transfected with GFP-fusion constructs or empty vector. 24 h post-transfection, cells were collected, and an aliquot (10%) was saved as “whole cell lysate” (WCL). Nuclear extracts were prepared with the Nuclear Complex Co-IP kit (ACTIVE MOTIF, Carlsbad, CA, USA) following the manufacturer’s instructions and processed for Co-IP with GFP-Trap_MA (Chromotek, Planegg, Germany). Briefly, GFP-Trap beads were equilibrated in IP incubation buffer (high stringency) (ACTIVE MOTIF), and binding of the proteins was performed overnight at 4 °C in IP incubation buffer. Beads were then washed in IP wash buffer (high stringency) (ACTIVE MOTIF), and the bound proteins were eluted in 2X SDS-sample buffer (120 mM Tris/HCl pH 6.8; 20% glycerol; 4% SDS, 0.04% bromophenol blue; 10% β-mercaptoethanol). Immunoprecipitates were analyzed by immunoblotting with an anti-E2F1 antibody (as described above).

### 2.7. RNA Extraction, RT–PCR, and RT–qPCR

Total RNA was isolated using the RNeasy Mini Kit (QIAGEN, Hilden, Germany) according to the manufacturer’s instructions and then treated with DNase (Ambion, Thermo Fisher Scientific, Waltham, MA, USA). cDNAs were synthesized with Superscript IV RT cDNA synthesis kit (Life Technologies) using 500–1500 ng of total RNA. An aliquot of the RT reaction (1–2 μL) was then PCR-amplified (with GoTaq DNA Polymerase, Promega, Madison, WI, USA), whereas, for quantitative PCR (qPCR), cDNAs were amplified with QuantiTect SYBR Green PCR (QIAGEN) or iTaq Universal SYBR Green Supermix (Bio-Rad, Hercules, CA, USA) by using LightCycler 480 (Roche). Target transcript levels were normalized to those of reference genes. The expression of each gene was measured in at least three independent experiments. All primers are listed in Supplementary Table S1. All PCR products were verified by sequencing.

### 2.8. RNA-seq and Analysis of Differentially Expressed Genes (DEGs)

To identify genes whose mRNA expression changed between control and Nova2 depleted moEC [28], we generated raw read counts using vast-tools v.1.1.0 [37] and log2 fold change (log2 FC) and adjusted *p*-values (between control and depletion) using DESeq2 v.1.0.19, R version 3.4.3 [38]. This found 4037 up- and 4310 downregulated genes after Nova2 depletion in ECs (adjusted *p*-value < 0.01). Given not all of these genes could be experimentally validated, we decided to filter our differentially expressed genes to those with a log2 FC > 1 (or < −1) and adjusted *p*-value < 1 × 10^−12^ (Supplementary Table S2).

### 2.9. Functional Enrichment Analysis

Functional gene enrichment analyses were performed using DAVID web tool (https://david.ncifcrf.gov/) [39] by creating a gene list composed of all the genes that have positive expression counts (basemean ≥ 1) across our RNA-seq dataset, and using it as background, entries were ranked accordingly with their adjusted *p*-value with Benjamini correction (< 0.05). Gene ontology (GO) enrichment analysis was also performed using the Enrichr web tool (http://amp.pharm.mssm.edu/Enrichr/) [40,41]; “GO Biological Process 2018” was used for ontologies, and entries were ranked accordingly with their combined score. Enrichr web tool “ChEA_2016” (ChIP-Seq enrichment experiments database, 2016) and “TRANSFAC and JASPAR PWMs” were used to explore ChIP peaks and DNA binding motifs of DNA binding protein at the promoter of input genes, respectively.

Enrichr and Cytoscape plug-in ClueGO (version 2.3.5) [42] were employed to perform functional analysis exploiting Reactome pathway and both KEGG and Reactome pathways, respectively, on the pool of 1437 genes differentially expressed after Nova2 depletion in moEC; for the ClueGO analysis, we used the same background list of GO analysis. The following parameters for ClueGO analysis were set: level 3–8, k = 0.4, Min Number of Genes = 8, Min Percentage = 8%, *p*-value < 0.05 (Enrichment; Bonferroni Step down).

### 2.10. Identification of Nova2 Target Genes Implicated in Transcriptional Regulation

To identify Nova2-AS events with a role in transcriptional regulation, genes predicted to generate different protein isoforms and belonging to the GO categories “transcription factor complex”, “chromatin assembly or disassembly”, “chromatin remodeling”, “chromatin silencing”, “ligand-dependent nuclear receptor transcription coactivator activity”, “regulation of gene expression, epigenetic”, “histone binding”, “negative regulation of gene expression, epigenetic”, “SWI/SNF superfamily-type complex” [28] were considered (Supplementary Table S3).

## 3. Results

### 3.1. Nova2 is a Regulator of EC Transcriptome

In order to comprehensively identify genes with AS events modulated by Nova2 in the endothelium, we previously used RNA-seq (high-throughput sequencing of RNA) of stable Nova2 knockdown and control ECs derived from mouse embryos (moEC) (Supplementary Figure S1A). Through this analysis, we identified 365 AS events affected by Nova2 depletion in ECs [28]. Using gene ontology (GO) analysis, in addition to genes controlling cytoskeleton organization and cell adhesion, which are consistent with the phenotypes described for Nova2-depleted ECs [28], we also found a significant enrichment for genes involved in chromatin remodeling or encoding for components of transcription factor complexes, raising the possibility that Nova2-mediated AS could modulate expression, activity, or localization of transcriptional regulators. 

**Figure 1:**
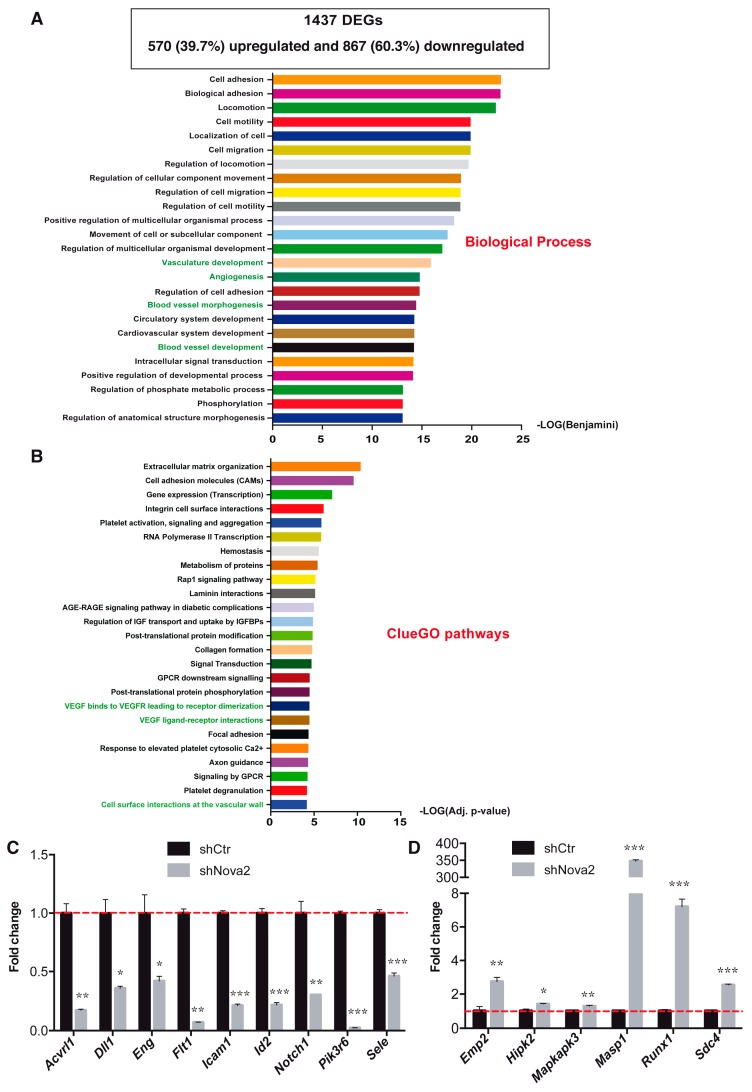
Differentially expressed genes (DEGs) in Nova2-depleted ECs. (**A**) The box shows the summary of DEGs with the number of downregulated and upregulated genes after Nova2 depletion in mouse endothelial cells (moEC); log2 fold changes (FC) < −1 (for downregulated genes), log2 FC > 1 (for upregulated genes); *p*-value < 10^−12^. Gene ontology (GO) analysis of DEGs in Nova2 knockdown moEC was performed by using the DAVID web tool. The first 25 terms of the GO category “Biological Process” are indicated (sorted by Benjamini corrected *p*-values < 0.05). GO terms in green font are relevant for EC biology. (**B**) Enrichment pathway analysis of DEGs in Nova2-depleted moECs performed by using ClueGO, a Cytoscape plugin. The first 25 pathways from both KEGG and Reactome repositories are shown (adjusted *p*-value < 0.05). Validation by RT-qPCR in Nova2-depleted moEC of (**C**) nine downregulated transcripts and (**D**) six upregulated mRNAs. The dotted red line indicates the expression level of control cells (shCtr) considered equal to 1. Each bar reports the mean ± SEM of three independent experiments. * *p* < 0.05, ** *p* < 0.01, *** *p* < 0.001.

Based on these observations, it is tempting to speculate that Nova2 could affect the steady-state mRNA levels of a number of genes in ECs. Therefore, to identify genes whose mRNA expression levels are altered upon Nova2 depletion (differentially expressed genes, DEGs), we re-analyzed our original RNA-seq data ([28]; PRJNA293346), comparing stably Nova2 knockdown and control moEC (strict filters: log2 fold change < −1 or > 1; adjusted *p*-value < 1 × 10^−12^). This found a total of 1437 DEGs after Nova2 knockdown, including 570 (39.7%) upregulated and 867 (60.3%) downregulated genes (Figure 1A, Supplementary Figure S1B, and Supplementary Table S2).

### 3.2. Nova2 Affects the Expression Levels of Genes Governing Key EC Functions

To explore the effect of Nova2 on the EC biology, we used GO and pathway analysis to find potential terms significantly enriched after Nova2 depletion (our 1437 DEGs). Interestingly, by using DAVID (https://david.ncifcrf.gov/) [39] and Enrichr (http://amp.pharm.mssm.edu/Enrichr/) [40,41], we found a significant enrichment for relevant GO terms in “Biological Process”, such as “vasculature development”, “angiogenesis”, “blood vessel development”, “negative regulation of sprouting angiogenesis”, and “endothelial cell development” (Figure 1A and Supplementary Figure S2A).

We then used Enrichr and the ClueGO Cytoscape plugin [42] to find the most enriched KEGG and Reactome pathways in which the Nova2-mediated DEGs were involved. Here we showed the enriched pathways below the adjusted *p*-value threshold <0.05 (Figure 1B and Supplementary Figure S2B). Among these pathways, we found “VEGF ligand-receptor interactions”, “VEGF binds to VEGFR leading to receptor dimerization”, and “cellular surface interactions at the vascular wall”.

In order to validate our RNA-seq data, we analyzed the expression levels of 15 genes selected since they encode for factors involved in angiogenesis and/or vascular development, according to GO terms or the literature [43,44,45,46,47]. By using RT-qPCR with RNA extracted from control and stable Nova2 knockdown moEC, we found that *Acvrl1*, *Dll1*, *Eng*, *Flt1*, *Icam1*, *Id2*, *Notch1*, *Pik3r6*, and *Sele* were downregulated (Figure 1C), while *Emp2*, *Hipk2*, *Mapkapk3*, *Masp1*, *Runx1*, and *Sdc4* were upregulated (Figure 1D). In addition, we also confirmed the Nova2-dependent expression of these DEGs by using RNA, extracted from stable Nova2 overexpressing moEC (Supplementary Figure S2C,D).

Collectively, beside the AS changes that we have previously identified [28], our analysis indicated that Nova2 also regulated the expression levels of several genes orchestrating key EC functions, thus suggesting a multilayered impact of Nova2 regulation in the vascular endothelium.

### 3.3. DEGs are Enriched for Ppar-γ Target Genes

Nova proteins can directly control mRNA expression levels by promoting AS events associated with the introduction of PTCs (premature translation-termination codons), which in turn activate nonsense-mediated mRNA decay (AS-NMD) [34]. Surprisingly, we found that only a small fraction of genes that changed their mRNA expression levels in moEC knockdown for Nova2 also changed their AS in the same ECs (29 out of 1437; 2% of the DEGs) (Supplementary Figure S3), suggesting the existence of indirect mechanisms through which Nova2 regulates steady-state mRNA levels in ECs. In particular, we found that 19 Nova2 splicing target genes identified in ECs [28] encoded for transcription factors or chromatin remodeling factors (see Materials and Methods and Supplementary Table S3). Since their mRNA expression levels did not change in Nova2-depleted moEC, we hypothesized that Nova2-mediated AS could generate variants of these proteins with different DNA binding activity, altered nuclear localization, or diverse ability to interact with co-factors. As a consequence, this could result in changes in steady-state mRNA levels of their target genes in ECs. To investigate this further, we used the ChIP enrichment analysis database (ChEA_2016; Enrichr [40,41]) to determine if our DEGs were enriched for target genes of transcriptional regulators with altered AS upon Nova2 knockdown in moEC. This analysis showed significant enrichment for genes that are the target of peroxisome proliferator-activated receptor-gamma (Ppar-γ or Pparg) and transcription factor 12 (Tcf12) (Supplementary Table S4). We decided to restrict our analysis to Ppar-γ since i) it has a well-characterized function in ECs [48]; ii) our Enrichr analysis found that a greater number of DEGs were targets of Ppar-γ (36.4%) compared to Tcf12 (11.8%).

**Figure 2:**
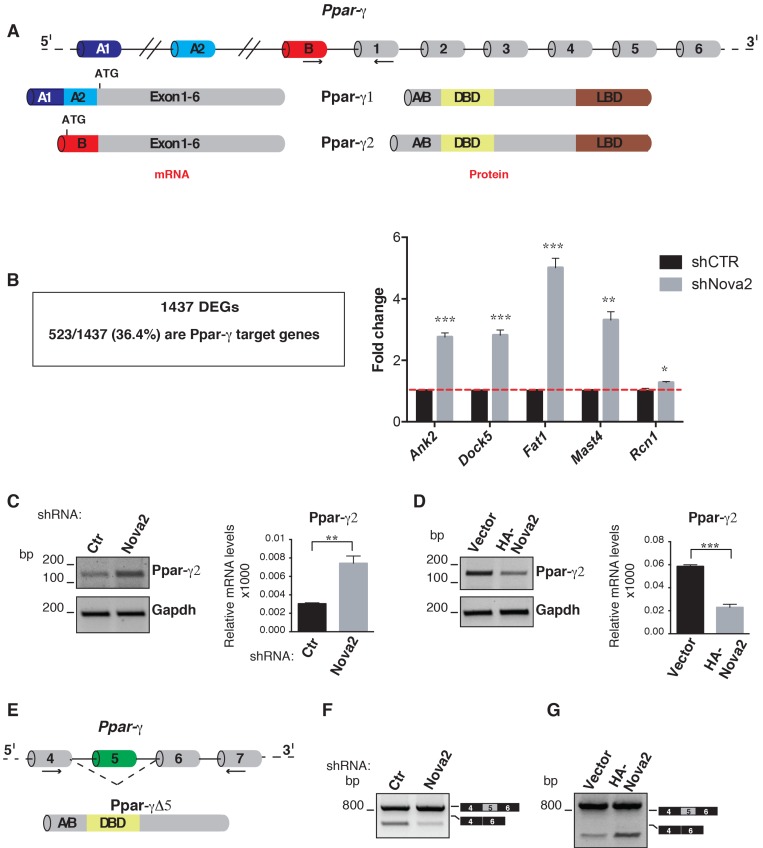
Nova2 (neuro-oncological ventral antigen 2) affects the splicing of Ppar-γ (peroxisome proliferator-activated receptor-gamma) and expression of its target genes in ECs (endothelial cells). (**A**) Several *Ppar-γ* mRNAs are generated by alternative transcription start sites and alternative splicing (AS) of different exons in the 5′ terminal region (for simplicity, only the first exons A1, A2, and B were schematized). *Ppar-γ1* mRNA contains exons A1 and A2, spliced together with exons 1–6, whereas, in *Ppar-γ2*, exon B is present instead of exon A1 and A2. The upstream ATG in exon B determinates the inclusion of 28 (mouse) or 30 (human) amino acids at the N-terminal of Ppar-γ2 protein. Boxes = exons; thin lines = introns. A/B = ligand-independent transactivation domain; DBD = DNA binding domain; LBD = ligand-binding domain. Arrows indicate primers used in RT-PCR reactions in C and D. (**B**) A total of 523 out of 1437 (36.4%) of DEGs were identified as Ppar-γ target genes in ChEA_2016 (adjusted *p*-value < 0.05) (left). Increased mRNA expression of five selected Ppar-γ targets was confirmed by RT-qPCR with RNA extracted from Nova2-depleted moEC (right). The dotted red line indicates the expression level of control cells (shCtr) considered equal to 1. (**C**) Expression of *Ppar-γ2* mRNA was evaluated by RT-PCR (relative to *Gapdh*) and RT-qPCR (relative to *Ubb*) in Nova2-depleted moEC. (**D**) Expression of *Ppar-γ2* mRNA was evaluated by RT-PCR and RT-qPCR in moEC overexpressing HA-tagged Nova2. (**E**) Skipping of *Ppar-γ* exon 5 determinates a shift in the open reading frame with the formation of a premature stop codon that generates the shorter Ppar-γ∆5 isoform lacking the LBD. (**F**) RT-PCR analysis of the AS profile of the *Ppar-γ* exon 5 in Nova2-depleted moEC and (**G**) in Nova2 overexpressing moEC. In all histograms, data indicate means ± SEM calculated from three independent experiments (*n* = 3); * *p* < 0.05, ** *p* < 0.01, *** *p* < 0.001. In each diagram, black arrows show the annealing position of the primers used in RT-PCR reactions.

Ppar-γ is a transcription factor able to drive specific gene expression programs upon stimulation with its ligand. Although Ppar-γ was initially identified for its roles in adipose tissue and lipid metabolism [49], additional analyses demonstrated that it is also an important regulator of EC biology in angiogenesis and during vasculature development and homeostasis [48]. In particular, the Ppar-γ pathway has been found to influence EC migration and proliferation, as well as the production of angiogenic factors by ECs [48]. Moreover, disruption of the Ppar-γ pathway has a critical role in a number of cardiovascular diseases [50]. Interestingly, several Ppar-γ isoforms, generated by a combination of the diverse transcription start sites and splicing of different exons encoding the N-terminus of the protein, have been described [51,52]. For example, Ppar-γ2 differs from Ppar-γ1 for the inclusion of an alternative first exon (exon B) encoding for 28 (mouse) or 30 (human) amino acids in the N-terminal ligand-independent activation domain (A/B domain) (Figure 2A), which is subjected to post-translational modifications (such as phosphorylation and SUMOylation) [53,54] and is able to interact with a number of cofactors, including the acetyltransferases CREB binding protein (CBP) and p300 [55]. Interestingly, common but also distinct functions of Ppar-γ1 and Ppar-γ2 have been reported [56,57], thus suggesting that the relative abundance of the different Ppar-γ variants could determine the overall biological effect of the Ppar-γ pathway in specific cellular or developmental contexts.

We found that a large fraction of genes displaying altered mRNA steady-state levels in Nova2-depleted moEC showed significant enrichment for Ppar-γ in the ChEA_2016 database (523 out of 1437; 36.4% of DEGs) in line with them being Ppar-γ target genes (Figure 2B and Supplementary Table S4). Changes in the expression levels of selected Ppar-γ targets were validated by using RT-qPCR with RNA extracted from control and Nova2 knockdown moEC (Figure 2B) or Nova2 overexpressing moEC (Supplementary Figure S4A). GO analysis revealed significant enrichment for regulators of angiogenesis and/or vascular development among these Ppar-γ targets, as exemplified in Supplementary Figure S4B.

We analyzed the AS profile of the Ppar-γ gene in ECs with altered Nova2 expression levels. As shown in Figure 2C, reduced Nova2 expression in moEC resulted in an increased level of the Ppar-γ2 mRNA isoform, and the opposite effect was observed in Nova2 overexpressing moEC (Figure 2D). By using SFmap (http://sfmap.technion.ac.il/) [58,59], we identified putative Nova2 binding sites (YCAY) in the intronic regions flanking Ppar-γ exon B (Supplementary Figure S5A), suggesting that the Ppar-γ pre-mRNA is a direct Nova2 target. Interestingly, we also identified putative Nova2 binding sites in Ppar-γ exon 5 (Supplementary Figure S5B), an AS exon whose skipping was recently shown to generate a short Ppar-γ isoform lacking the ligand-binding domain (Ppar-γ∆5), thus acting as a dominant-negative isoform reducing Ppar-γ activity (Figure 2E) [60]. Since ligand-mediated Ppar-γ activation promotes skipping of Ppar-γ exon 5, it has been proposed that this AS event could function as a negative feedback loop to regulate Ppar-γ activity [60]. This observation prompted us to validate the Nova2-mediated AS of Ppar-γ exon 5 in ECs. As shown in Figure 2F, we found that Nova2-depleted moEC displayed reduced expression of Ppar-γ∆5 mRNA. Conversely, forced expression of Nova2 in moEC increased Ppar-γ∆5 transcript (Figure 2G). As an additional control, we depleted the moEC of Rbfox2—another AS regulator with a role in endothelium [61]. While the AS profile of a known Rbfox2 target gene [62] was affected by its silencing in ECs (Supplementary Figure S6A,B), splicing of Ppar-γ pre-mRNA was not influenced in these treated cells (Supplementary Figure S6C,D), further supporting a Nova2-specific effect. Based on our data, we proposed that Nova2-mediated AS regulation generated different Ppar-γ isoforms, whose spatial and/or temporal expression could affect Ppar-γ functions that, through the modulation of target gene expression, could be important to orchestrate EC biology.

### 3.4. Nova2 Controls the Nuclear Localization of E2F Dimerization Partner 2

**Figure 3:**
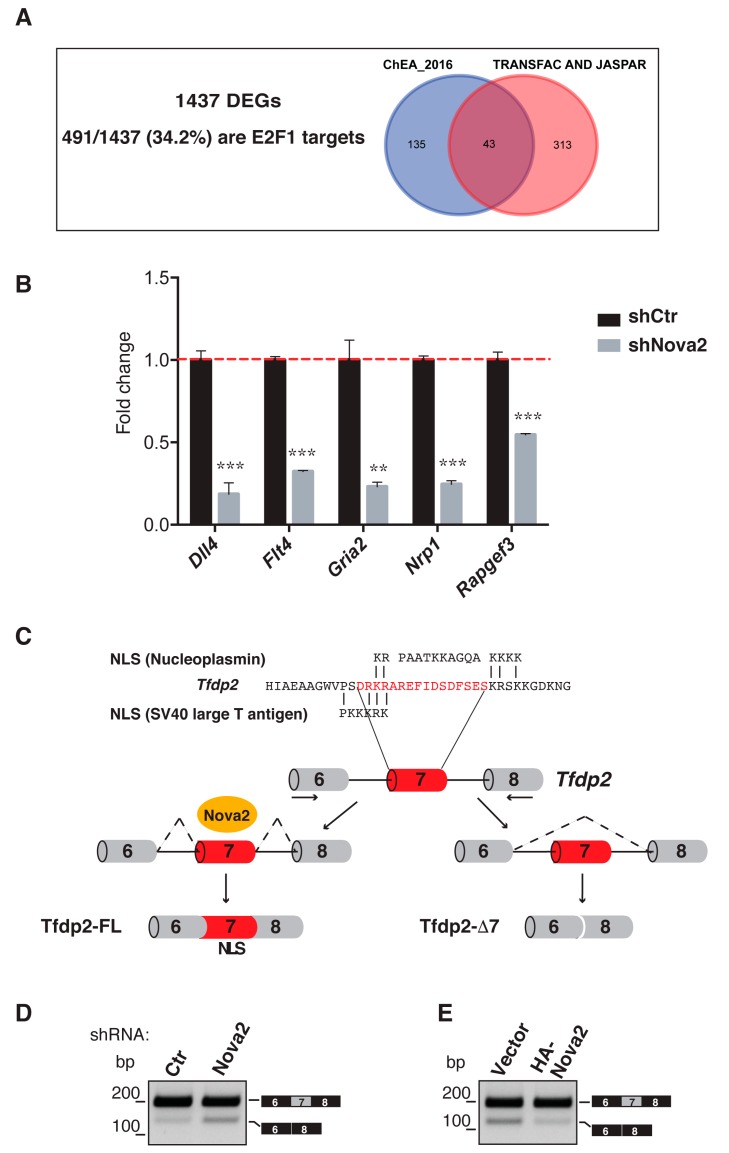
Nova2 affects the expression of E2F1 target genes and regulates AS of *Tfdp2* exon 7. (**A**) 491 out of 1437 (34.2%) of DEGs were identified as E2F1 target genes by using databases available on Enrichr web tool (ChEA_2016; TRANSFAC and JASPAR) (adjusted *p*-value < 0.05). (**B**) mRNA expression levels of five selected E2F1 targets were confirmed by RT-qPCR in Nova2-depleted moEC. The dotted red line indicates the expression level of control cells (shCtr) considered equal to 1. (**C**) Top. Comparison of the amino acids encoded by the mouse *Tfdp2* exon 6 (final part), exon 7 (of 48 nt), and exon 8 (first part) with SV40 large T antigen nuclear localization signal (NLS) (bottom) and with nucleoplasmin bi-partite NLS. Bottom. Schematic representation of the *Tfdp2* mouse genomic region comprising the AS exon 7 (red box). Grey boxes = constitutive exons; thin lines = introns; dashed arrows indicate primers used in RT-PCR reaction. The presence of Nova2 stimulates the inclusion of exon 7 in *Tfdp2* mRNA encoding for the Tfdp2-FL variant; the absence (or low level) of Nova2 promotes skipping of exon 7 and the production of the Tfdp2-∆7 protein. (**D**) AS profile of *Tfdp2* exon 7, as determined by RT-PCR in mouse moEC knockdown for Nova2 and in (**E**) moEC overexpressing Nova2.

One additional possibility through which Nova2 could control the steady-state mRNA levels is its ability to regulate AS of genes encoding for interactors/regulators of transcription factors. In line with this, we found that one of the Nova2 splicing target genes encodes for E2F dimerization partner 2 (Tfdp2), a member of the Tfdp family that forms heterodimers with E2F family proteins, thus stimulating E2F-dependent transcription [63]. E2F proteins (E2F1–E2F8) were initially characterized for their ability to control the expression of genes involved in cell proliferation, differentiation, and apoptosis [63]. However, recent findings show that some E2F members function in a number of physiological processes in addition to cell cycle control [64,65,66,67]. Interestingly, our Enrichr analysis found that our DEGs were significantly enriched for E2F1 target genes present in the ChEA_2016 database (Figure 3A and Supplementary Table S5). Moreover, our DEGs were also significantly enriched for E2F1 binding motifs by using other databases available on Enrichr (TRANSFAC and JASPAR) (Figure 3A and Supplementary Table S5. Altogether, we found that 491/1437 (34.2%) of our DEGs were E2F1 targets (Figure 3A). Changes in the expression levels of selected E2F1 targets were also verified by using RT-qPCR with RNA extracted from control and Nova2 knockdown moEC (Figure 3B) or Nova2 overexpressing moEC (Supplementary Figure S7A). Remarkably, by using GO analysis, we found that several of these E2F1 targets encode for regulators of angiogenesis and/or vascular development, as exemplified in Supplementary Figure S7B. In line with our observation, several works described the pivotal role of E2F1 in vascular biology [68,69,70,71,72]. Accordingly, E2F1 binding sites are present in several genes encoding for factors involved in angiogenesis (such as FLT-1, KDR, and Angiopoietin 2), and E2F1 binding to these promoters is induced in ECs upon VEGF stimulation [72].

Our previous RNA-Seq data [28] suggested that Nova2 was able to promote inclusion on the mature mRNA of the *Tfdp2* exon 7 that encoded for 16 amino acid residues containing a putative nuclear localization signal (NLS) [73] (Figure 3C). Hence, it is tempting to speculate that Nova2 could affect Tfdp2 localization and, as a consequence, the function of the Tfdp2-E2F1 heterodimer. We confirmed by RT-PCR that depletion of Nova2 in moEC increased skipping of the Tfdp2 exon 7 (Figure 3D), whereas the forced expression of Nova2 increased its inclusion (Figure 3E). In contrast, the depletion of Rbfox2 had no effect on AS of *Tfdp2* exon 7, supporting a specific Nova2 effect (Supplementary Figure S6E). As previously reported, the direction of the observed AS changes in the *Tfdp2* pre-mRNA was consistent with the position of Nova2 binding sites (YCAY) in intron 7 (Supplementary Figure S5C). 

**Figure 4:**
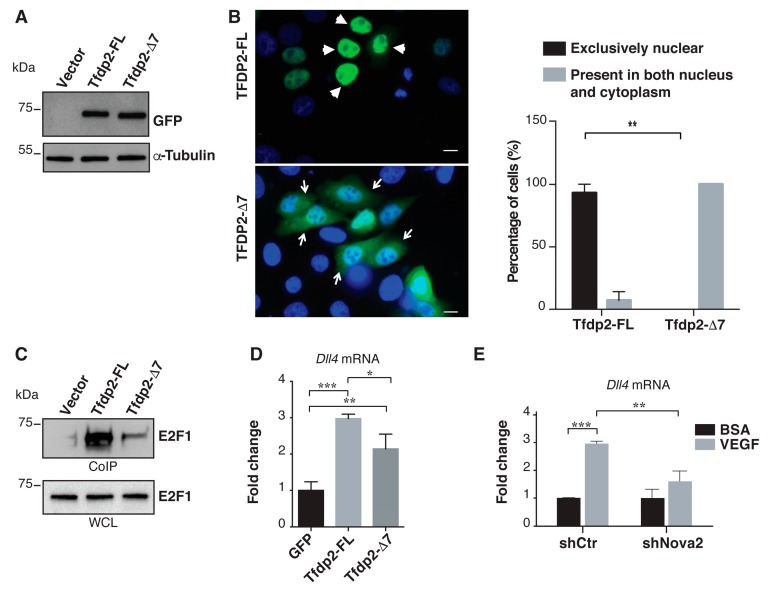
Cellular localization of Tfdp2 isoforms and their interaction with E2F1. (**A**) Expression of GFP-tagged Tfdp2 isoforms (Tfdp2-FL and Tfdp2-∆7) or the empty vector (Vector) was verified with an anti-GFP antibody on lysates from HeLa cells. (**B**) Representative images of HeLa cells transfected with the GFP-tagged Tfdp2 isoforms (scale bar 10 μm). Arrowheads show the nuclear localization of Tfdp2-FL, while arrows show the nuclear and cytosolic localization of Tfdp2-∆7. The histogram on the right shows the quantitation of the cellular localization of the two Tfdp2 isoforms. Values represent means ± SEM from at least five different fields per experiment in each condition (n of independent experiments = three). Comparisons between experimental groups were done with two-sided Student’s t-test; ** *p* < 0.01. (**C**) HeLa cells were transfected with the GFP-tagged Tfdp2 isoforms (or the empty vector), and their interaction with E2F1 was tested after separation of the nuclear fraction and co-immunoprecipitation (Co-IP) with an anti-GFP antibody. Whole cell lysate (WCL) and immunoprecipitates were analyzed by immunoblotting with an anti-E2F1; a representative Co-IP experiment out of four is shown. (**D**) RNAs from HeLa cells transfected with GFP-tagged Tfdp2 isoforms (or empty vector) were extracted from FACS-sorted GFP-positive cells and analyzed by RT-qPCR for the expression level of *Dll4* mRNA. Each bar represents the normalized mean expression level ± SD of three independent transfections. Comparisons among experimental groups were done with ANOVA test and Tukey’s multiple comparisons test. Expression levels of a cell transfected with the empty vector (GFP) were considered equal to 1. (**E**) RT-qPCR for the expression of *Dll4* mRNA in control and Nova2 knockdown moEC treated with VEGF (100 ng/mL for 24 h). Expression levels of control cells (shCtr) and Nova2 knockdown (shNova2) treated with BSA were considered equal to 1. Each bar reports the mean ± SD of three independent experiments. * *p* < 0.05, ** *p* < 0.01, *** *p* < 0.001.

To test if the AS regulation of *Tfdp2* exon 7 could affect Tfdp2 nuclear localization, we generated two GFP-tagged Tfdp2 protein isoforms with (Tfdp2-FL) or without the 16 amino acids (Tfdp2-∆7) encoded by exon 7 (Figure 4A), and we analyzed their intracellular distribution after transfection in HeLa cells. Importantly, as shown in Figure 4B, we found that Tfdp2-FL is mainly localized in nuclei, whereas Tfdp2-∆7 failed to efficiently accumulate in nuclei and was present both in the nucleus and in the cytoplasm. This result suggested that the nuclear accumulation of Tfdp2-FL could allow interaction with its physiological partner, E2F1. To test this hypothesis, we transfected HeLa cells with the GFP-tagged Tfdp2 isoforms and evaluated their ability to co-immunoprecipitate with the endogenous E2F1 protein. As shown in Figure 4C, we found that Tfdp2-FL was more associated with E2F1 compared to Tfdp2-∆7. In addition, we found that the expression level of Dll4, a well-characterized E2F1 target gene involved in angiogenesis [74], was significantly increased in HeLa cells overexpressing Tfdp2-FL compared to Tfdp2-∆7 (Figure 4D). As reported before, E2F1 binding sites are present in several genes encoding angiogenesis regulators, and E2F1 binding to these promoters is induced in ECs upon VEGF stimulation [72]. Since we found that E2F1 binding sites were present in the *Dll4* promoter (see our Enrichr analysis in Supplementary Table S5), we decided to extend our results in ECs. As shown in Figure 4E, we found that VEGF stimulation induced the expression of *Dll4* mRNA in control moEC, whereas *Dll4* induction was significantly reduced upon Nova2 knockdown in line with its requirement for *Dll4* activation by VEGF. Collectively, our results promoted Nova2 as an AS regulator of a nuclear localization signal whose final effect was to influence the subunit composition of a heterodimeric transcription factor and, as a consequence, its transcriptional activity.

## 4. Discussion

Regulation of gene expression programs plays a key role in angiogenesis, a very complex process in which a sequential series of events, such as EC sprouting, lumen formation, and tubulogenesis, are controlled through the coordinated action of a number of transcription factors [75]. Similarly, an intricate network of interactions between these transcription factors, the majority of which are not EC specific, occur in vascular development and during arterial, venous, and lymphatic EC differentiation [75]. However, several studies demonstrated that morphological and functional changes involved in vascular biology require reprogramming of gene expression that is only in part accomplished at the transcriptional level. Indeed, epigenetic changes and post-transcriptional mechanisms can influence EC gene expression programs in physiological and pathological conditions [76,77,78].

Alternative splicing (AS) is an important post-transcriptional mechanism of gene expression that expands the human proteome and tightly controls cell identity during tissue and organ development [1]. Notably, AS deregulation contributes to multiple aspects of tumor establishment, progression, and resistance to treatments [4,5,6,7,79]. However, despite the established role of AS in several human processes, its impact on angiogenesis (one of the hallmarks of cancer) has not been widely investigated. In this context, we previously demonstrated that the AS factor Nova2 was an important post-transcriptional regulator of angiogenesis and was required for correct vascular development [28]. The importance of Nova2 in angiogenesis is also emphasized by recent data showing that it is upregulated in cancer vasculature, including ovarian cancer (OC) and colorectal carcinoma, while no Nova2 expression is detectable in other cell types present in the tumors [30,31]. Of note, high Nova2 expression correlates with shorter overall survival of OC patients [31], supporting its potential as a prognostic marker.

Here we found that Nova2, in addition to AS, regulated steady-state gene expression. Intriguingly, differentially-expressed genes (DEGs) identified in Nova2 knockdown ECs were enriched for genes encoding for angiogenesis and vascular development factors, providing new insights into the involvement of Nova2 in EC functions. The observation that in neuronal cells, Nova proteins control steady-state mRNA levels through AS-coupled NMD (AS-NMD) [34] prompted us to investigate if Nova2-mediated AS regulation explains the DEGs identified in ECs. However, we found that only a small fraction of our DEGs were likely associated with the ability of Nova2 to regulate AS-NMD, suggesting that differential and cell type-specific mechanisms of Nova2 action regulated distinct gene expression programs. Nevertheless, mRNAs containing PTCs can be quickly degraded by NMD and might not be detectable by RNA-seq unless NMD is inhibited [80]. Future work is thus required to fully evaluate the impact of Nova2-mediated AS-NMD in ECs.

By searching indirect mechanisms through which Nova2 could modulate mRNA steady-state levels in the endothelium, we identified 19 genes encoding for transcription factors or chromatin remodeling complexes among the Nova2 splicing targets in ECs [28]. This is in line with the very interesting observation that genes encoding for transcription factors have the highest rate of AS, thus promoting the AS mechanism as a major determinant of transcription factor diversity [81]. We hypothesized that the Nova2-mediated AS of transcriptional regulators could generate novel protein variants with distinct functions, and we focused our attention on Ppar-γ, a transcription factor initially identified as a key regulator of adipogenesis, glucose homeostasis, and lipid metabolism [82], but over time found to play an important role also in the vascular system [48]. Also, altered Ppar-γ functions can lead to cardiovascular problems, such as atherosclerosis, restenosis, and hypertension [50]. We found that in ECs, Nova2 regulated the usage of *Ppar-γ* exon B (an alternative first exon), which is part of the A/B domain, the latter subjected to post-translational modifications [53,54] and involved in Ppar-γ interactions with co-regulators [55]. The role of Nova proteins in alternative first exon regulation is not unexpected since it has been already reported for Pasilla, the Nova homolog in *Drosophila melanogaster* [83]. Hence, it could be interesting to determine if Nova2 acts directly on *Ppar-γ* exon B or if its effect is a consequence of functional correlation between AS process and transcription [84]. Noteworthy, Nova2 also promoted in ECs the production of the *Ppar-γ∆5* transcript encoding for a dominant-negative isoform able to negatively modulate Ppar-γ-induced target genes [60]. From a wider viewpoint, naturally occurring dominant-negative (or attenuated) variants are a common regulatory mechanism, helping to reduce the *trans*-activation capability of their cognate factors [85,86,87,88]. Interestingly, a large fraction of genes, which displayed altered mRNA steady-state levels in Nova2 knockdown ECs (36.4% of DEGs), overlapped with Ppar-γ targets and were enriched for GO terms relevant for angiogenesis and vascular development. We found that Ppar-γ2 expression levels were increased, whereas Ppar-γ∆5 (encoding for a dominant-negative isoform reducing Ppar-γ activity) was reduced in Nova2 knockdown ECs. Since Ppar-γ is a well-characterized transcriptional activator [89], we decided to validate Ppar-γ targets upregulated in Nova2 silencing ECs. However, we also found downregulation of several Ppar-γ targets upon Nova2 depletion, suggesting the possibility that in ECs, Ppar-γ could interact with corepressors and directly silence the expression of some of its targets, as previously described [90]. On this basis, we speculated that the Nova2-mediated AS regulation of *Ppar-γ* pre-mRNA could contribute to the overall Ppar-γ activity in ECs. Recent data showed that Nova2 was also expressed in the adipose tissue, where it controlled an AS program, contributing to thermogenesis [91]. Considering the important role of Ppar-γ in adipogenesis, our findings opened an interesting scenario in which Nova2 could contribute to Ppar-*γ* regulation of adipose tissue functions.

In the present study, we also defined a role for the Nova2-mediated AS regulation of the *Tfdp2* gene, which encodes for the E2F dimerization partner 2 (Tfdp2). We found that Nova2 promoted the inclusion of *Tfdp2* exon 7 in the mature mRNA, leading to the production of a Tfdp2 protein variant that accumulates in the nucleus. Beyond the control of the cell cycle, E2F transcription factors have been described to function in a variety of cellular processes, including apoptosis, angiogenesis, adipogenesis, and cell migration [65]. Notably, putative E2F1 binding sites are present in many genes involved in angiogenesis and, for some of them, the recruitment of E2F1 to their promoters is stimulated by VEGF [72], strongly supporting an E2F1 involvement in EC biology. In line with this, we found that 491/1437 (34.2%) of our DEGs were E2F1 target genes. In addition, by performing a GO analysis, we found that these E2F1 targets were significantly enriched for relevant GO terms, including “vascular development, “blood vessel development”, “blood vessel morphogenesis”, and “angiogenesis”.

We focused on E2F1 ability to function as a transcriptional activator [92], and we validated E2F1 targets that were downregulated upon Nova2 depletion in ECs, since in these cells, Tfdp2-∆7 (that fails to efficiently locate in the nucleus) was more expressed compared to control ECs. Interestingly, similar to Ppar-γ, several E2F1 targets were also regulated in the opposite direction (that is upregulated). Importantly, a direct connection between Ppar-γ and E2F1-dependent transcription has been known for a long time [93]. Thus, it is tempting to speculate that a number of E2F1 target genes could be indirectly upregulated in our ECs as a consequence of the increased activity of Ppar-γ. In line with this, we also found that a high number of our DEGs were common targets of both Ppar-γ and E2F1 transcription factors (Supplementary Figure S7C).

Of note, among DEGs characterized by the presence of putative E2F1 binding sites in their promoters, we identified *Dll4. Dll4*, whose mRNA level was reduced in Nova2 knockdown ECs and increased in Nova2 overexpressing ECs, encodes for a Notch ligand that regulates tip cell formation in angiogenesis, as well as vessel sprouting and branching during vascular development [94,95]. Dll4/Notch signaling is also implicated in tumor angiogenesis [96]. In particular, Dll4 represents a potential biomarker and therapeutic target in OC, a type of tumor characterized by strong angiogenesis and showing Nova2 upregulation selectively in the tumor vasculature [31]. Increased expression of Dll4 in OC is associated with poor overall patient survival [97,98], whereas Dll4 silencing in combination with VEGF inhibition was found potentially able to improve the outcome of OC therapy [98].

Taken together, our findings supported a novel role of Nova2 in ECs by modulating the activity of a number of transcription factors, thus integrating AS decisions in the intricate network of gene expression programs involved in EC biology.

## Supplementary Materials

The following are available online at https://www.mdpi.com/2073-4409/8/12/1498/s1, Figure S1: Nova2 knockdown in ECs and Volcano plot showing gene expression changes after Nova2 depletion in ECs, Figure S2: GO and cellular pathways analysis of Nova2-mediated DEGs, Nova2 overexpression in ECs, and differentially expressed genes after Nova2 upregulation, Figure S3: Comparison between DEGs and Nova2-regulated genes in ECs, Figure S4: Nova2 affects the expression of Ppar-γ target genes that are enriched for regulators of EC biology, Figure S5: Identification of putative Nova binding sites in *Ppar-γ* and *Tfdp2* genes, Figure S6: Depletion of *Rbfox2* in ECs does not affect AS of *Ppar-γ* and *Tfdp2* pre-mRNAs, Figure S7: Nova2 modulates the expression of E2F1 target genes that are enriched for regulators of EC functions, Table S1: Primers used in RT-PCR, RT-qPCR reactions, and cloning experiments, Table S2: Differentially Expressed Genes (DEGs) after Nova2 depletion in ECs, Table S3: Genes encoding for transcriptional regulators for which Nova2-mediated AS is predicted to generate different protein isoforms from [28], Table S4: Lists of DEGs that are targets of peroxisome proliferator-activated receptor-gamma (Ppar-γ) or transcription factor 12 (Tcf12), Table S5: Lists of DEGs that are targets of E2F1.
