# Supplementary material for: Gene Expression Profiles Controlled by the Alternative Splicing Factor Nova2 in Endothelial Cells
Source: Cells. 2019 Nov 23;8(12):1498. doi: 10.3390/cells8121498 (PMC6953062; doi:10.3390/cells8121498)
Supplement: Supplementary file 1 [file cells-08-01498-s001.zip › Supplementary Figures_Proof_2019_DP.pdf]

cells

MDPI

Supplementary Materials: Gene Expression Profiles Controlled by the Alternative Splicing Factor Nova2 in Endothelial Cells

Elisa Belloni, Anna Di Matteo, Davide Pradella, Margherita Vacca, Christopher D.R. Wyatt, Roberta Alfieri, Antonio Maffia, Simone Sabbioneda and Claudia Ghigna

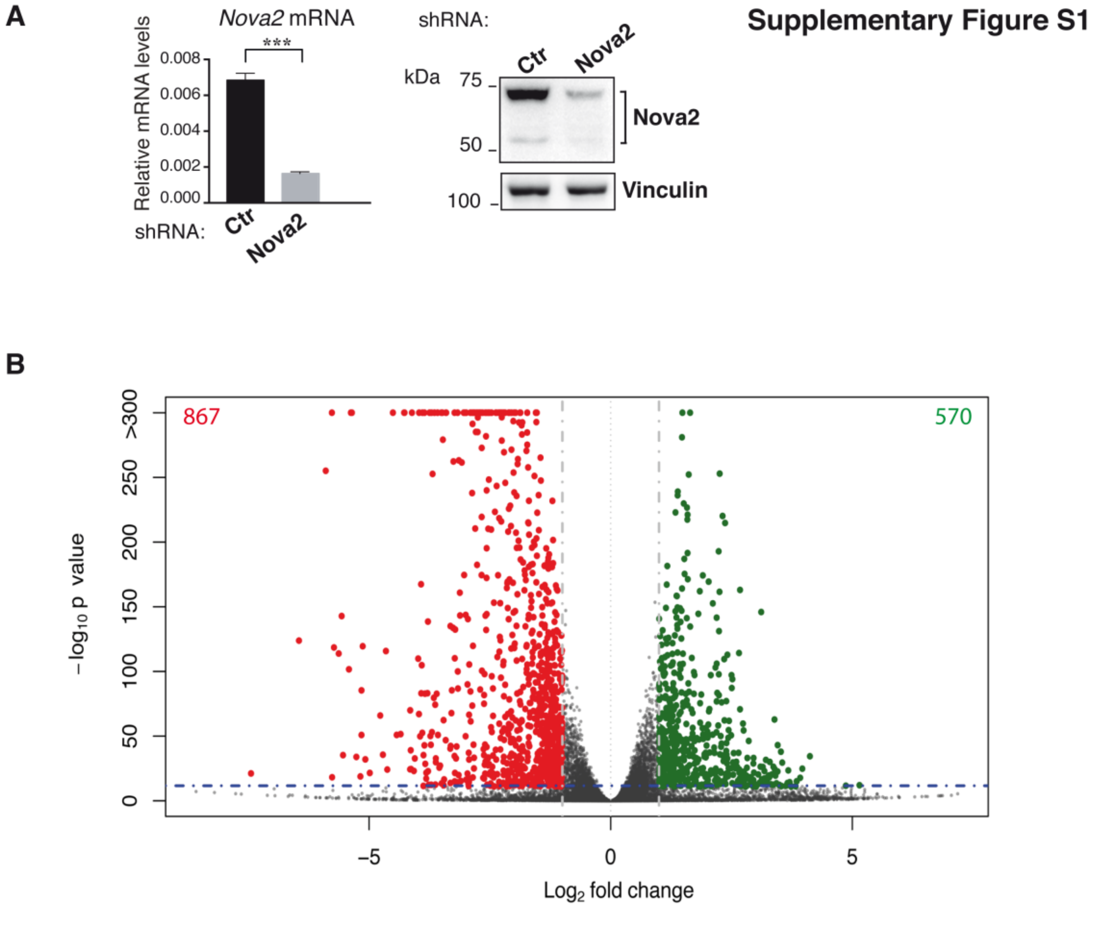

Figure S1. Nova2 knockdown in ECs and Volcano plot showing gene expression changes after Nova2 depletion in ECs. **(A)** Validation by RT-qPCR (relative to *Ubb*) and immunoblotting of Nova2 expression levels in moEC transduced with an shRNA against *Nova2* or with a control shRNA. Each bar reports mean ± SEM of three independent experiments. ***p<0.001. **(B)** Volcano plot: each point represents a gene, with the x axis showing the log2 FC of gene expression between control and Nova2 depletion and y axis showing the DESeq2 -log10 adjusted p-values. Red and green point show down- and upregulated genes respectively. Grey points show genes that were considered not significant, either with adjusted p-value>1e^{-12} and/or absolute log2 FC<1.

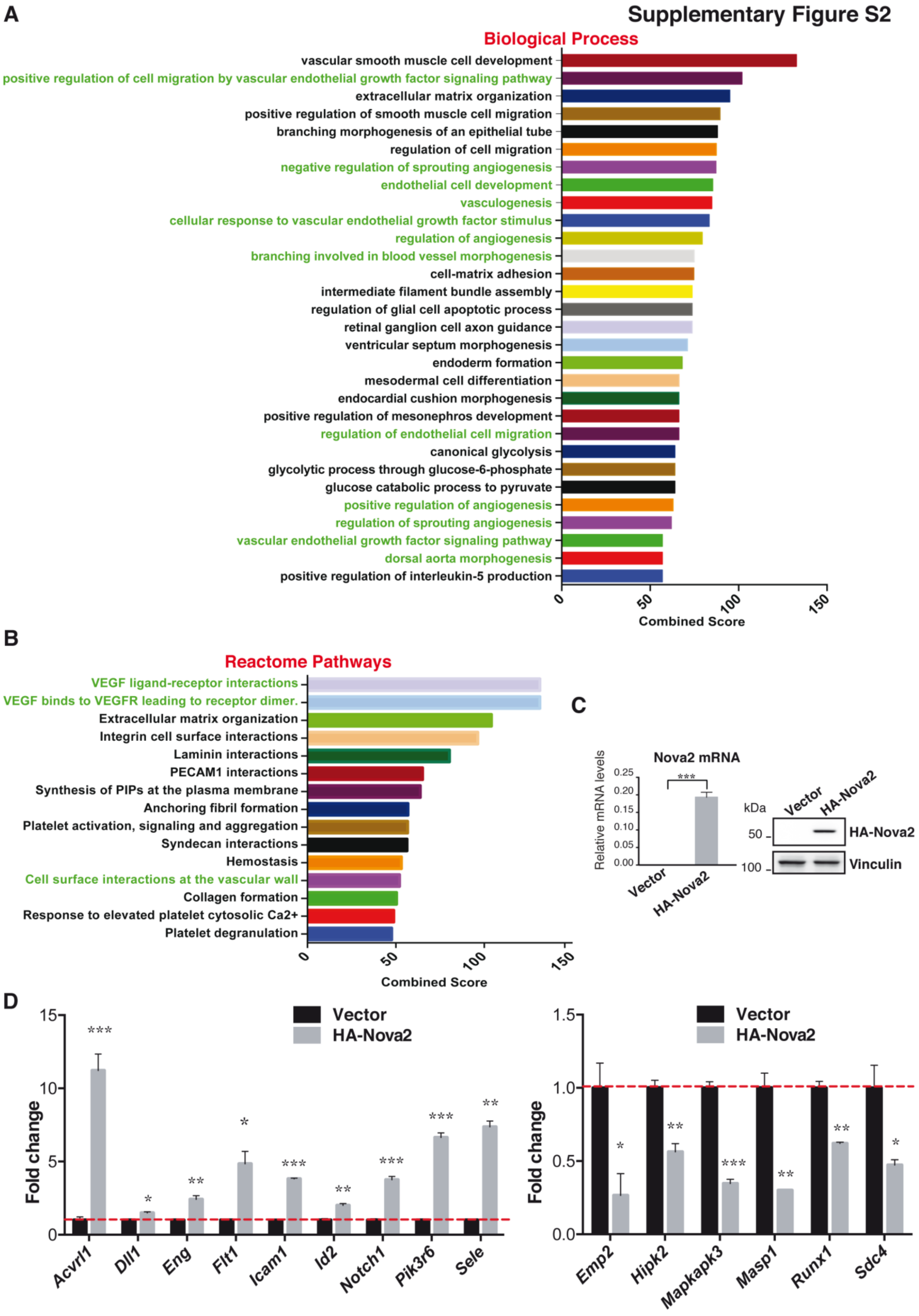

Figure S2. GO and cellular pathways analysis of Nova2-mediated DEGs, Nova2 overexpression in ECs and differentially expressed genes after Nova2 upregulation. **(A)** GO analysis of genes showing altered expression levels in Nova2 knockdown moEC by using the Enrichr web tool. The first 30 terms of the GO categories

“Biological Process” are indicated (based on Combined Score). **(B)** Analysis of cellular pathways by using the Enrichr web tool (based on Combined Score). **(C)** Upregulation of HA-tagged Nova2 in moEC was verified by RT-qPCR (relative to *Ubb*) and immunoblotting with an anti-HA antibody. **(D)** Analysis in Nova2 overexpressing moEC of the mRNA levels of selected genes encoding for factors involved in angiogenesis, vascular development or EC functions. The dotted red line indicates the expression level of control cells (empty vector) considered equal to 1. Error bars indicate means ± SEM calculated from three independent experiments (n = 3). *p<0.05, **p<0.01, ***p<0.001.

Supplementary Figure S3

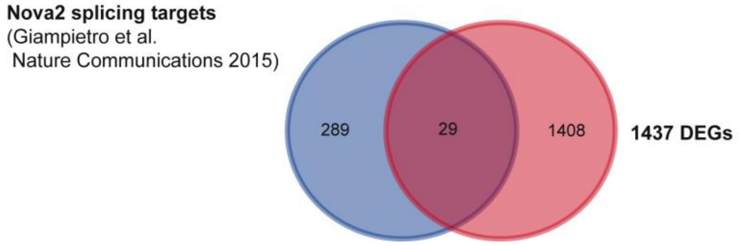

Figure S3. Comparison between DEGs and Nova2-regulated genes in ECs. The Venn diagram shows the overlap between genes changing their steady-state mRNA levels in Nova2 knockdown ECs (1437 DEGs) and genes regulated by Nova2 at the level of splicing in the same cells (318 genes for a total of 365 AS events;[28]).

Supplementary Figure S4

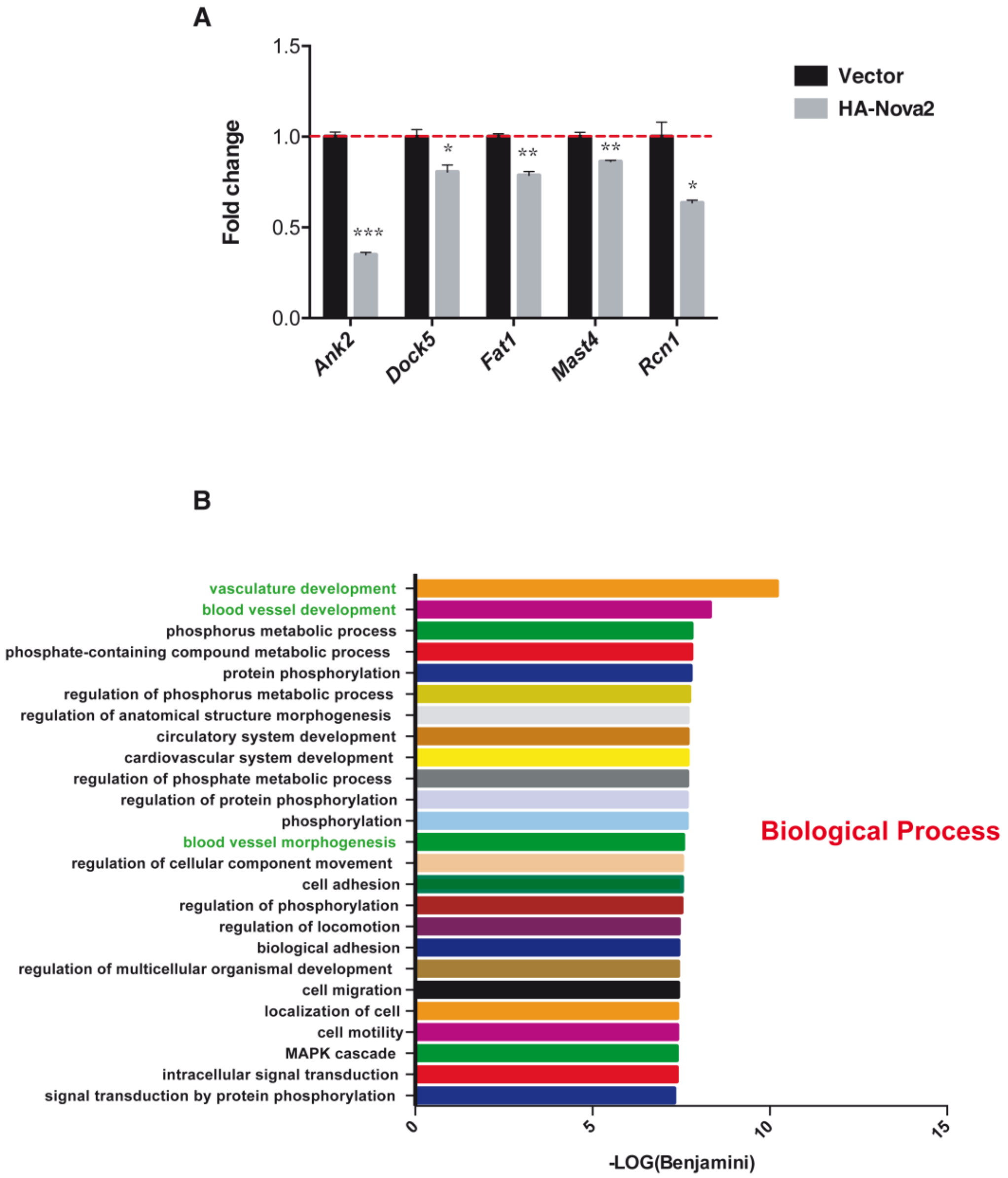

Figure S4. Nova2 affects the expression of Ppar-γ target genes that are enriched for regulators of EC biology. **(A)** Expression levels of five selected Ppar-γ target genes determined by RT-qPCR in Nova2 overexpressing moEC. The dotted red line indicates the expression level of control cells (empty vector) considered equal to 1. **(B)** GO analysis of Ppar-γ target genes, showing altered expression levels in Nova2 knockdown moEC, by using the DAVID web tool. The first 25 terms of the GO category "Biological Process" are indicated (sorted by benjamini corrected p-values <0.05). In green GO terms relevant for angiogenesis and vascular development.

Supplementary Figure S5

A

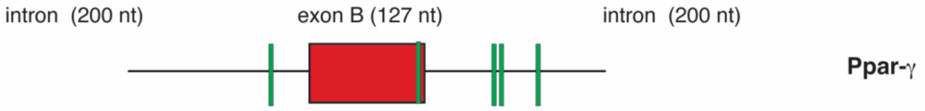

GACAGCATTATGACACACCATTTTGTCACAACTGGCTCTCAGTCAGGACAGTGCCAGCCAATTCAGGCCTGATTCTTTCTGTGTTTATTCCCACCTCTCCCAAATATTTGAAAACTGGTG
TCTTGACTTATTAGCATATTCATAAGCTCGATGACCATAAGCCTTTTTCCTTTAACCAACCAATCTTTTGCAAGACATAGCAAAACACCAGTGTGAATTACAGCAAATCTCTGTTTTATG
CTGTTATGGGTGAAACTCTGGGAGATTCTCCTGTTGACCCAGAGCATGGTGCCTTCGCTGATGCACTGCCTATGAGCACTTCACAAGGTAAAGTCCATTCCTGATGTTGCTGCAAGGGAT
GCTTCGGGCACTCATGTGTGGGTGAAATTGCATGTTTGTTTTCCACATGTATTCATATAGTCTATTAATCCTTTGATCATTTTTATGGTGATCACTTATAAATGTATTCAGAACCTTCAG
CATAGAACCCTAATCTAATTTCTATTTTATCTCTGCTCATGGCTTCC

B

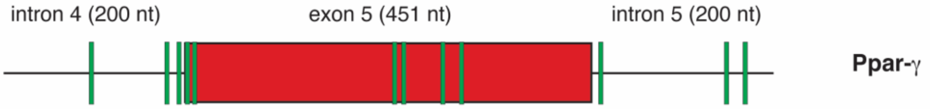

AAGAAAAAAAGAGCCAAGTTGATAAAATGTTTGCACACAGCAGATCAATGATAAATAGGAAGAGCCTCTCTCCTGTTTAAGTGCCTTCATCTTTCACAGTTTTCCTCAGCAAGGAAATAC
ATATATAAAATAGTCTTCAGGACTAGGGTGATGCTCTCTCAGAACAAGGAACTCAGCCACTTTCCCTTTTTCATTTGCAGCCATTTGTCATCTACGACATGAATTCCTTAATGATGGGAG
AAGATAAAATCAAGTTCAAACATATCACCCCCCTGCAGGAGCAGAGCAAAGAGGTGGCCATCCGAATTTTTCAAGGGTGCCAGTTTCGATCCGTAGAAGCCGTGCAAGAGATCACAGAGT
ATGCCAAAAATATCCCTGGTTTCATTAACCTTGATTTGAATGACCAAGTGACTCTGCTCAAGTATGGTGTCCATGAGATCATCTACACGATGCTGGCCTCCCTGATGAATAAAGATGGAG
TCCTCATCTCAGAGGGCCAAGGATTCATGACCAGGGAGTTCCTCAAAAGCCTGCGGAAGCCCTTTGGTGACTTTATGGAGCCTAAGTTTGAGTTTGCTGTGAAGTTCAATGCACTGGAAT
TAGATGACAGTGACTTGGCTATATTTATAGCTGTCATTATTCTCAGTGGAGGTAAGATTCATCTTTTGATTTTTGTCAAAGAAGATGGGATTGTGGTGGAATTGTTGGTGGGATTGTGTC
TCGGTGCTCTGTGCTCCTTTTTACTGACTGTGCGTGTTGTGTAGAACATGCCAATGGCACAGTGCCGTGAGCATGTCACTCCCCAGGCTGAGAACCATAGGCTTGCAGAGTGAAGGTGGT
GGGGATCCCAG

C

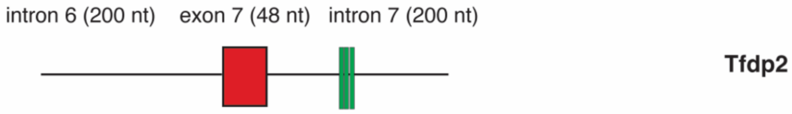

AAATCTATACTGTTTTTCTCCCCAAGTATTCATTGAACTTATGCCAATTTTGTATTATTATATTGTCATATCTTTATCTTTGTGAAAATAAGTTTTCTTTTGTATACATTCAGTGTAGAG
TGAGACAGTTTATAATAAATTATTTAAGAAATAGAGTTGTGTTTATGTGTTGTGCTTAAGCTATCTTTTTGTCTTTTTAGTGATAGAAAACGAGCTAGAGAATTTATAGACTCTGATTTT
TCAGAAAGGTGAGTATAAGTCTGCTTTGTTTCATATTTCACTATGGAAAGAGGGGAGTTTCCTTTGCAACTTTAACTTTATTTAGTTTCTACCATCATTTCATTCTACACTGAGATTTTC
AATTCTCTGATTTGCAGTTTCAGTAGATAGCTTGTGACATTTGTCATTTAGGCCCCTCTCTTGTTTTGGGGAAGACAGTGTTCTTCCA

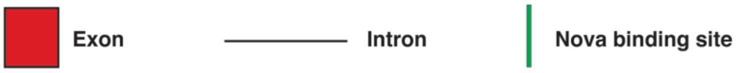

Figure S5. Identification of putative Nova binding sites in *Ppar-γ* and *Tfdp2* genes. The mouse genomic regions comprising *Ppar-γ* exon B (**A**) *Ppar-γ* exon 5 (**B**) and *Tfdp2* exon 7 (**C**) plus 200 nt of upstream and 200 nt downstream intronic sequences, were analyzed with the SFmap program (http://sfmap.technion.ac.il/). Putative Nova binding sites (green underlined) were identified by using the following parameters: score 0,9-1 and p≤0.005. Red = exonic sequence; black = intronic sequence. The schematic representation of the genomic region and the position of Nova binding sites is also depicted on top of each gene sequence.

Supplementary Figure S6

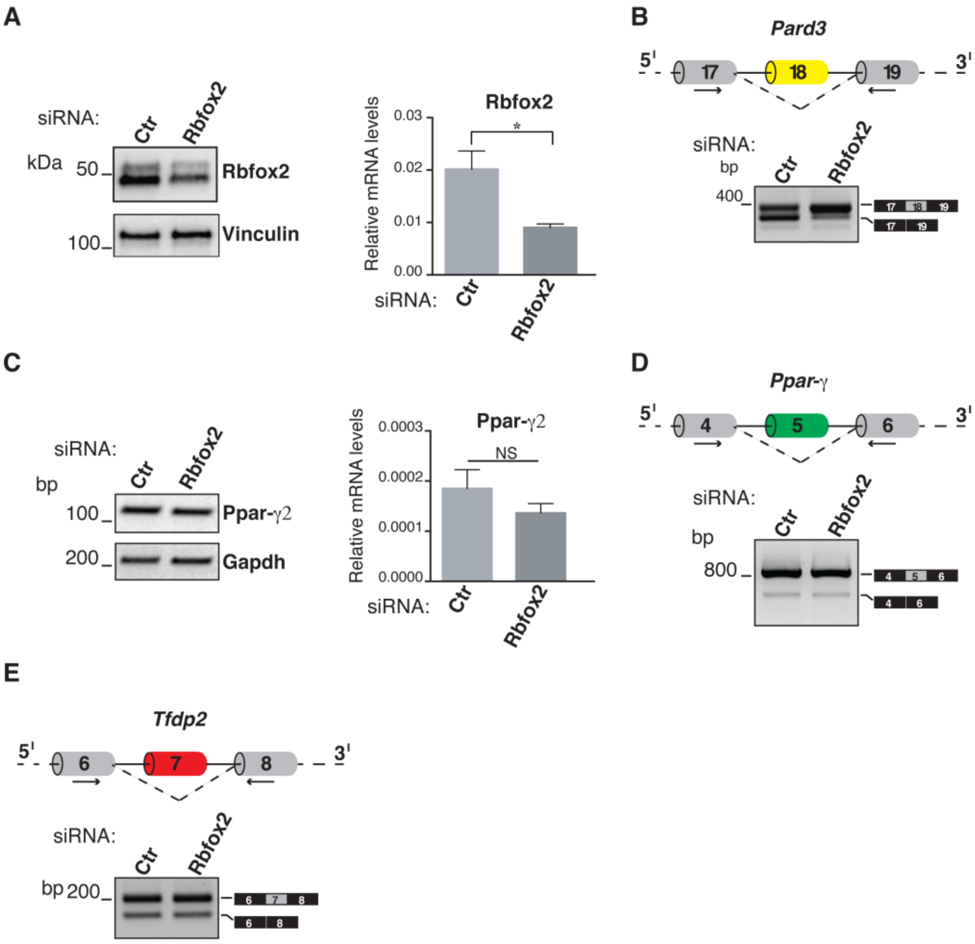

Figure S6. Depletion of *Rbfox2* in ECs does not affect AS of *Ppar-γ* and *Tfdp2* pre-mRNAs. **(A)** Rbfox2 mRNA and protein levels after its knockdown in mouse moEC. **(B)** Analysis by RT–PCR of the AS of *Pard3* (a known Rbfox2 target) upon *Rbfox2* silencing in moEC. **(C)** Expression of *Ppar-γ2* mRNA was evaluated by RT-PCR (left) and RT-qPCR (right) in *Rbfox2* depleted moEC. **(D)** RT-PCR analysis of the AS profile of *Ppar-γ* exon 5 and **(E)** *Tfdp2* exon 7 in *Rbfox2* knockdown moEC. Error bars indicate means ± SEM calculated from four independent experiments (n = 4, *p<0.05). In each diagram, black arrows show the annealing position of the primers used in RT-PCR reactions.

Supplementary Figure S7

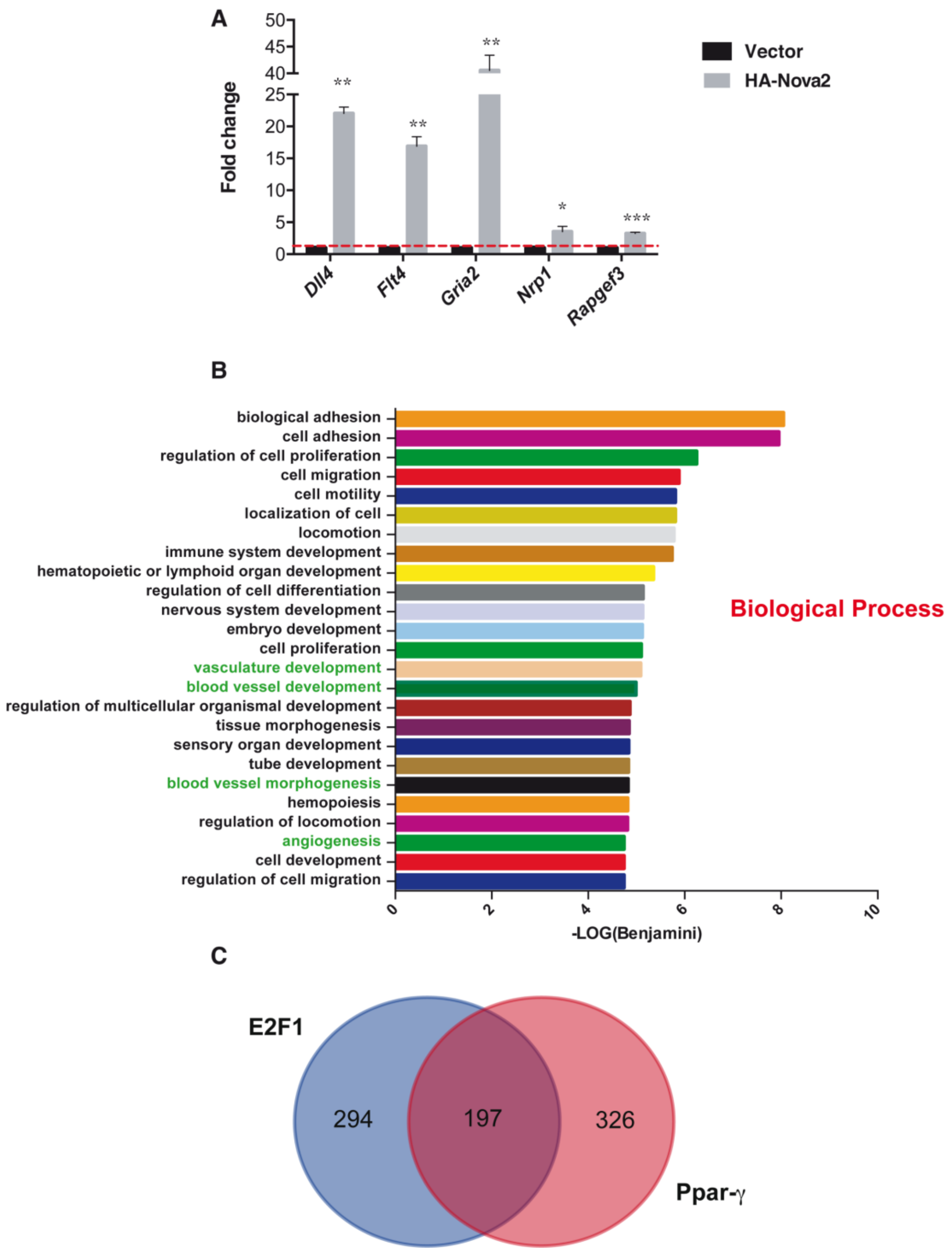

Figure S7. Nova2 modulates the expression of E2F1 target genes that are enriched for regulators of EC functions. **(A)** Expression levels of five selected E2F1 targets by RT-qPCR in Nova2 overexpressing moEC. The dotted red line indicates the expression level of control cells (empty vector) considered equal to 1. Error bars indicate means ± SEM calculated from three independent experiments (n=3), *p<0.05, **p<0.01, ***p<0.001. **(B)** GO analysis of E2F1 targets, which expression levels are modulated upon Nova2 silencing in ECs, by using the DAVID web tool. The first 25 terms of the GO category "Biological Process" are indicated (sorted by benjamini corrected p-values <0.05). In green GO terms that are relevant for angiogenesis and vascular development. **(C)** DEGs that are common targets of both Ppar-γ and E2F1 transcription factors.

Table S1. Primers used in RT-PCR, RT-qPCR reactions and cloning experiments.

**PRIMERS USED IN RT-PCR**	
**PRIMER NAME**	**SEQUENCE 5'-3'**
Ppar-γ_mouse_exon B_for	TTCTCCTGTTGACCCAGAGCA
Ppar-γ_mouse_exon 1_rev	TCCACAGAGCTGATTCCGAAG
Ppar-γ_mouse_exon 4_for	TGCCTTGCTGTGGGGATGT
Ppar-γ_mouse_exon 7_rev	CAGCAGGTTGTCTTGGATGT
Gapdh_mouse_for	TCAAGAAGGTGGTGAAGCAGG
Gapdh_mouse_rev	ACCAGGAAATGAGCTTGACAAA
Tfdp2_mouse_for	CCGCAATGGTCACTCAGACT
Tfdp2_mouse_rev	TGTGCCTTTCCGCTGAACTT
Pard3_mouse_for	CTCGCTTTTCAACGGGAAGG
Pard3_mouse_rev	TCCATGCCTTCGTCGTCATC
**PRIMERS USED IN RT-qPCR**	
**PRIMER NAME**	**SEQUENCE 5'-3'**
Ubb_mouse_for	CCGGCAAGCAGCTAGAAGAT
Ubb_mouse_rev	ATTGGGGCAAGTGGCTAGAG
Acvrl1_mouse_for	GGCCTTTGGCCTAGTGCTAT
Acvrl1_mouse_rev	GGGGTCATTGGGTACCATGT
Dll1_mouse_for	ATCCGATACCCAGGTTGTCT
Dll1_mouse_rev	CAGGAACATGTGTAGCTCCC
Eng_mouse_for	TGTTCCTGGTCCTCGTTTCG
Eng_mouse_rev	GCCCAGTCGAGGATCTGTTT
Flt1_mouse_for	GAAAAGTCCGTGTCCTCGCT
Flt1_mouse_rev	TGATTGTTGGCCGAGGGATG
Icam1_mouse_for	GCCAATTTCTCATGCCGCAC
Icam1_mouse_rev	GTGTCGAGCTTTGGGATGGTA
Id2_mouse_for	AGCAAAGTACTCTGTGGCTAAAT
Id2_mouse_rev	TCTCCTGGTGAAATGGCTGAT
Notch1_mouse_for	ATTTTAGCGACGGCCACTGT
Notch1_mouse_rev	CAGCACAGTCTAGGCCATCC
Pik3r6_mouse_for	ACCTCACCAAATGCTGCTTC
Pik3r6_mouse_rev	GGATACTGGATGTCCGGAAGG
Sele_mouse_for	GCAGAGTTTCACGTTGCAGG
Sele_mouse_rev	CTGTGGCGCAGATAAGGCT
Emp2_mouse_for	TCTGGAGAGTGTGCACCAAC
Emp2_mouse_rev	GAGAGGATCATGGTGGCCTG
Hipk2_mouse_for	CCAAATTTGTGCCCGACCTG

Hipk2_mouse_rev	GGACAGCATTTTCCATCCGC
Mapkapk3_mouse_for	TAATGCGGGACATTGGCACT
Mapkapk3_mouse_rev	TGGCAAAGCCAAAATCGGTG
Masp1_mouse_for	CAAAGCCAGGAGCTCACAGA
Maps1_mouse_rev	CACGGTATGGGCTGAAACCT
Runx1_mouse_for	CTGCTCCGTGCTACCCACT
Runx1_muose_rev	AGTAGTTTTCATCGTTGCCTGC
Sdc4_mouse_for	TGACTTTGAGCTCTCGGGTT
Sdc4_mouse_rev	GGGCTCAATCACTTCAGGGAA
Ank2_mouse_for	ACATACCCCCAGAGACGGT
Ank2_mouse_rev	CTCCTCCTTCTCTGTGCCATC
Dock5_mouse_for	ACTCCCAAAGCCACGAGAAC
Dock5_mouse_rev	CATACGGTTTGCTTTTGGGGG
Fat1_mouse_for	CTTCCAACTCGCCCTCAGAC
Fat1_mouse_rev	GTATGGCTGGGAGGGCTTTT
Mast4_mouse_for	GCTTAGTGAGGATGGGAAGCA
Mast4_mouse_rev	CGAGGTTGGACGTCTCCG
Rcn1_mouse_for	CCTCAGGACTACGACCATGC
Rcn1_mouse_rev	AGGTCTTCCCCGTAGTTGGT
Nova2_mouse_for	TGCTGTCCACAGCTTTATCG
Nova2_mouse_rev	GCTCCTCCCTTACCGATGAT
Nova2_human_for	CAGCTTTATTGCCGAGAAGG
Nova2_human_rev	ACCCATGCTCCTGACTGTTC
Ppar-γ_mouse_exon B_for	TTCTCCTGTTGACCCAGAGCA
Ppar-γ_mouse_exon 1_rev	TCCACAGAGCTGATTCCGAAG
Dll4_mouse_for	TGAGAAGCCAGAGTGTCGAA
Dll4_mouse_rev	GCTGGGTGTCTGAGTAGGCT
Flt4_mouse_for	CTTGGTGTCCATTCCCGGC
Flt4_mouse_rev	AACAGTTGAGTGGGCACCCG
Gria2_mouse_for	TACGGCAGAAGGAGTAGCCA
Gria2_mouse_rev	TTTCATGGTGTCGCAAGGCT
Nrp1_mouse_for	CTACAGCTGGACCAACCACA
Nrp1_mouse_rev	CCTCCTGTGAGCTGGAAGTC
Rapgef3_mouse_for	ATTCTGCCGGTGATGTCGTT
Rapgef3_mouse_rev	TCATGCACTTCCTGTGGGTC
Rbfox2_mouse_for	CCAACAAGAAGATGGTCACG
Rbfox2_mouse_rev	TGTTGATGCCTCCTCTTCCT
Rplp0_human_for	ATGCCCAGGGAAGACAGGGCG
Rplp0_human_rev	CGAAGGGACATGCGGATCTGCTGC
Dll4_human_for	CAACCAGGGGGCCAACTATG
Dll4_human_rev	CAAGGGTTACGGGCACAGTC
**PRIMERS USED IN CLONING EXPERIMENTS**	

**PRIMER NAME**	**SEQUENCE 5'-3'**
Tfdp2_mouse_PGFPC1_BglII_for	GGAAGATCTATGACGGCAAAAAATGTTGG
Tfdp2_mouse_PGFPC1_KpnI_rev	CGGGGTACCTTATTCTGGGGAGGAGGGAT

Table S3. Genes encoding for transcriptional regulators for which Nova2-mediated AS is predicted to generate different protein isoforms from [28].

**GO Term**	**Nr. Genes**	**Genes**
transcription factor complex	8	Dcp1a, Hax1, Nfya, Taf4b, Tcf12, Tfdp2, Tfe3, Trrap
chromatin assembly or disassembly	3	Baz2a, H2afy, Smarcd3
chromatin remodeling	6	Baz1a, Baz2a, Chd8, Nasp, Scmh1, Smarcd3
chromatin silencing	3	Baz2a, Phf8, Ubr2
ligand-dependent nuclear receptor transcription coactivator activity	3	Ncoa1, Pparg, Smarcd3
regulation of gene expression, epigenetic	4	Baz2a, H2afy, Phf8, Ubr2
histone binding	4	Baz2a, Chd8, Nasp, Phf8
negative regulation of gene expression, epigenetic	3	Baz2a, Phf8, Ubr2
SWI/SNF superfamily-type complex	3	Baz1a, Smarcd3, Trrap
